# *Closteroviridae*: the beginning

**DOI:** 10.3389/fmicb.2014.00014

**Published:** 2014-02-03

**Authors:** Moshe Bar-Joseph

**Affiliations:** S. Tolkowsky Laboratory, Virology Department, Agricultural Research Organization, Plant Protection InstituteBet Dagan, Israel

**Keywords:** closteroviridae, virus taxonomy, history, characterization, viruses

Forty years ago, an unusual name—closterovirus—was coined for an unusual group of elongated plant viruses (Bar-Joseph and Hull, 1974). This essay reflects my personal encounter with these viruses between 1966 and 1986, a period that could be considered the beginning of the emergence of the *Closteroviridae* as an exciting complex virus family.

The first two viruses assigned to this group, *Beet yellows virus* (BYV) and *Citrus tristeza virus* (CTV), have significant economic importance and, therefore, attracted considerable biological and epidemiological attention long before their molecular characterization. The seminal paper by Kitajima et al. (1964) reporting the association of long thread-like particles (TLP) with tristeza-expressing plants triggered much interest on the possibility of using those particles for diagnostic purposes. In 1966, I embarked on a Ph.D. project supervised by Prof. Gad Loebenstein that aimed to purify the TLP and develop a serological assay to be eventually used for the rapid detection of CTV-infected trees in case of an emerging epidemic. Isolating the long, thin, fragile TLP from woody tissue in the absence of a bioassay for quantitative estimation of the outcome of the numerous clarification, concentration and purification steps was a difficult and frustrating task. Indeed after almost three years, my attempts were still mostly unsuccessful. In retrospect, allowing me to continue the project at that stage was both remarkably generous and far-sighted. Improvements in TLP purification, including (i) the finding that young bark of only certain citrus species is the best source of TLP, (ii) the use of a careful extraction procedure and precipitation of TLP using polyethylene glycol, and (iii) the use of different combinations of buffers for extraction and resuspension allowed us to obtain sufficiently purified TLP particles to establish their viral-like composition and biophysical nature (Bar-Joseph et al., 1972), the infectivity of which was demonstrated by Garnsey et al. (1977).

The capable assistance of Mr. J. Cohen with electron microscopic analysis comparing the concentrations of TLP following the endless purification steps enabled me to complete my Ph.D. thesis in November 1972, almost six years after starting. I then took a post-doc position at the John Innes Institute (JII), Norwich. Shortly after my arrival, I realized that, 3 years earlier, a JII Ph.D. candidate had begun working on the characterization of BYV, but that work had been discontinued due to difficulties in obtaining purified BYV preparations. I asked for permission to use the CTV purification procedure for the isolation of BYV and, to the delight of the JII director, the late Prof. Roy Markham, with a few minor modifications this method was highly successful.

Working in cooperation with Roger Hull, we obtained sufficient amounts of BYV for the biophysical and molecular characterization of the virions and determined the sizes of their major coat protein subunits and RNA (Bar-Joseph and Hull, 1974). From these experiments we inferred that CTV and BYV shared not only similar particle structures, as revealed earlier by electron microscopy, but also closely similar RNA to coat protein mass ratios, thus providing direct virological support for their classification in a distinct taxonomic group.

However, because of the considerable variation in length, we suggested that the new group should be named *Closter virus* (*closter* is Greek for thread) to reflect the common morphological characteristic of its members, in contrast to previous groups of elongated plant viruses whose names were derived from their type members. Later analysis of *Carnation necrotic fleck virus* (CNFV), which shares several common biological features with BYV, further indicated the considerable degree of molecular and cytopathological similarity among closteroviruses as reported in the first review of this group (Bar-Joseph et al., 1979), which after a third of a century remains the main source of information on the biology of these viruses.

In 1980, Dr. Allan Dodds found large amounts of distinct dsRNA molecules in CTV-infected citrus tissues. The extension of his analyses to plants infected with BYV and CNFV revealed considerable similarities in the amounts of dsRNA they contained, as well as in their dsRNA profiles (Dodds and Bar-Joseph, 1983). It is interesting to note that years later the accumulation of large amounts of dsRNAs in plants infected by other members of the *Closteroviridae* was instrumental for their molecular cloning and genome characterization, despite the absence of purified virions.

The first phase of my *Closteroviridae* work ended in 1986 with the realization that, despite advances in serological and molecular detection methods, the natural spread of CTV in Israel had developed into an epidemic that could not be controlled by eradication. Fortunately, most prevalent isolates induced only mild symptoms and even now, almost 30 years later, CTV remains a minor disease problem regardless of earlier projections that giving up on eradication would destroy the local citrus industry (see Bar-Joseph et al., 1989).

The other reasons for considering this period as the beginning has to do with considerable advances in *Closteroviridae* research mostly by new groups of molecular virologists whose excellent work is summarized in the present Frontiers series. Looking back, despite the difficulties and disappointments, I feel a great deal of satisfaction from friendships shared through these years with numerous dear colleagues and students that, unfortunately, space limits prevent listing.
